# Supplementary material for: A Systematic Review and Meta-Analysis of the Prognostic Impact of Pretreatment Fluorodeoxyglucose Positron Emission Tomography/Computed Tomography Parameters in Patients with Locally Advanced Cervical Cancer Treated with Concomitant Chemoradiotherapy
Source: Diagnostics (Basel). 2021 Jul 14;11(7):1258. doi: 10.3390/diagnostics11071258 (PMC8304455; doi:10.3390/diagnostics11071258)
Supplement: Supplementary file 1 [file diagnostics-11-01258-s001.zip › Table S1.pdf]

Table S1. Search strategy

1. Medline search strategy

(("uterine cervical neoplasms"[MeSH Terms] OR ("uterine"[All Fields] AND "cervical"[All Fields] AND "neoplasms"[All Fields]) OR "uterine cervical neoplasms"[All Fields]) AND ("chemoradiotherapy"[MeSH Terms] OR "chemoradiotherapy"[All Fields])) AND ("positron emission tomography computed tomography"[MeSH Terms] OR ("positron"[All Fields] AND "emission"[All Fields] AND "tomography"[All Fields] AND "computed"[All Fields] AND "tomography"[All Fields]) OR "positron emission tomography computed tomography"[All Fields])

2. Embase search strategy

'uterine cervix tumor'/exp AND ('chemoradiotherapy'/exp OR 'chemoradiation' OR 'chemoradiotherapy' OR 'radiochemotherapy') AND ('computer assisted emission tomography'/exp)
