# Supplementary material for: A Systematic Review and Meta-Analysis of the Prognostic Impact of Pretreatment Fluorodeoxyglucose Positron Emission Tomography/Computed Tomography Parameters in Patients with Locally Advanced Cervical Cancer Treated with Concomitant Chemoradiotherapy
Source: Diagnostics (Basel). 2021 Jul 14;11(7):1258. doi: 10.3390/diagnostics11071258 (PMC8304455; doi:10.3390/diagnostics11071258)
Supplement: Supplementary file 1 [file diagnostics-11-01258-s001.zip › Table S2.pdf]

Table S2. Summary of prognostic results about pretreatment FDG PET parameters as a continuous variable.

Surname of first author	Parameters	OS			DFS		
		HR	95%CI	P-value	HR	95%CI	P-value
Carpenter[26]	SUV_{max}	1.019	0.970-1.062		1.028	0.986-1.066	
	SUV_{mean}	0.988	0.810-1.186		1.028	0.868-1.213	
	MTV	1.005	1.001-1.009	P<0.05	1.004	1.001-1.007	P<0.05
	TLG	1.065	1.003-1.123	P<0.05	1.051	0.998-1.099	
Hong[32]	SUV_{max}				1.04	0.99–1.08	
Calles-Sastre[27]	SUV_{mean}	1.06	0.98-1.14		1.03	0.96-1.09	
	MTV	1.55	1.10-2.19	P<0.05	1.55	1.10-2.19	P<0.05
	TLG	1.67	1.15-2.42	P<0.05	1.43	1.07-1.93	P<0.05
Scher[24]	SUV_{max}	1.03	0.95-1.12		1.03	0.96-1.11	
	SUV_{mean}	1.09	0.87-1.36		1.12	0.90-1.38	
	MTV	1.01	1.00-1.02	P<0.05	1.01	1.00-1.01	P<0.05
	TLG	1.001	1.00-1.01	P<0.05	1.001	1.00-1.01	
Liu[28]	SUV_{max}	1.03	0.93-1.13				
	MTV	1.01	1.00-1.01	P<0.05			
Oh[31]	SUV_{max}				0.999	0.922-1.082	
Chong[23]	SUV_{max}				3.1	1.32-7.28	P<0.05
	MTV				3.78	1.54-9.31	P<0.05
	TLG				3.5	1.49-8.21	P<0.05
