# Supplementary material for: A Systematic Review and Meta-Analysis of the Prognostic Impact of Pretreatment Fluorodeoxyglucose Positron Emission Tomography/Computed Tomography Parameters in Patients with Locally Advanced Cervical Cancer Treated with Concomitant Chemoradiotherapy
Source: Diagnostics (Basel). 2021 Jul 14;11(7):1258. doi: 10.3390/diagnostics11071258 (PMC8304455; doi:10.3390/diagnostics11071258)
Supplement: Supplementary file 1 [file diagnostics-11-01258-s001.zip › Table S3.pdf]

Table S3. Summary of prognostic results about FDG PET parameters during treatment.

Surname of first author	Parameters	OS			DFS		
		HR	95%CI	*P* value	HR	95%CI	*P* value
Carpenter [26]	SUV_{max}	1.019	0.970-1.062		1.062	0.985-1.142	
	MTV	1.011	1.004-1.018	P<0.05	1.009	1.002-1.015	P<0.05
	TLG	1.437	1.153-1.553	P<0.05	1.192	1.035-1.338	P<0.0
	SUV_{max}	1.13	0.98-1.30				
	MTV	1.01	1.00-1.01	P<0.05			
Scher[24]	SUV_{max}	1.872	1.236-2.853	P<0.05	1.601	1.217-2.106	P<0.05
	MTV	1.068	1.012-1.128	P<0.05	1.062	1.021-1.105	P<0.05
	TLG	1. 12	1.0001-1.023	P<0.05	1.01	1.003-1.017	P<0.05
