# Supplementary material for: A Systematic Review and Meta-Analysis of the Prognostic Impact of Pretreatment Fluorodeoxyglucose Positron Emission Tomography/Computed Tomography Parameters in Patients with Locally Advanced Cervical Cancer Treated with Concomitant Chemoradiotherapy
Source: Diagnostics (Basel). 2021 Jul 14;11(7):1258. doi: 10.3390/diagnostics11071258 (PMC8304455; doi:10.3390/diagnostics11071258)
Supplement: Supplementary file 1 [file diagnostics-11-01258-s001.zip › Table S4.pdf]

Table S4. Information about PET/CT technique and image analysis.

Author	Year	Technique	Fasting period	Dosage	Blood glucose levels	Interval time	CT scans	Slice thickness	MTV	TLG
Herrera[8]	2016	Discovery 690FX TOF	6h	3.5 MBq/kg	-	-	-	-	the FDG avid tumor volume measured bi-dimensionally at the longest diameter	SUV_{mean} multiplying MTV
Yilmaz[21]	2018	Discovery ST	6h	5-6 MBq/kg	<180mg/dl	60 minutes	140 kV and 80mA	3.75mm	40% of the SUV_{max}	SUV_{mean} multiplying MTV
Guler[15]	2018	Gemini TF 16	-	370-555 MBq (10-15mCi)	<150 mg dl	60 minutes	140 kV and 80 mA	4mm	equal to or greater than SUV of 2.525	SUV_{mean} multiplying MTV
Hong[17]	2016	Gemini TF 16	6h	370-555 MBq (10-15mCi)	<150 mg dl	60 minutes	140 kV and 80 mA	4mm	voxels with a SUV intensity of greater than 2.5 within the contouring margin	SUV_{mean} multiplying MTV
Chong[12]	2015	Reveal RT-HiREZ6-slice Ctapparatus/Discovery STE	6h	8.1 MBq/kg	<150 mg/dL	60 minutes	-	3mm/3.27mm	the mean SUV of the mediastinal background plus two standard deviations	SUV_{mean} multiplying MTV
Cima[14]	2017	GEDiscoverySTE	6h	3 to 5 MBq/kg	-	60 minutes	120 kV and 80 mA	3mm	-	-
Lucia[16]	2018	Philips Gemini/Siemens Biograph	4h	5 MBq/kg	<7 mmol/l	60 minutes	120 kV and 80 mA	-	-	-
Im[11]	2014	Biograph LSO	8h	444-740MBq (12-20mCi)	-	60 minutes	130 kV and 50 mA	4mm	-	-
Onal[18]	2015	Discovery-STE8	6h	370 to 555 MBq (10-15 mCi)	<150 mg/dL	60 minutes	140 Kv and 80 mA	5mm	-	-
Lee[19]	2020	Discovery STE	6h	5.5 MBq/kg	<150 mg/dL	60 minutes	140 kV and 30 to 170 mA	3mm		
Chong[12]	2017	Reveal RT-HiREZ6-slice Ctapparatus/Discovery STE	6h	8.1 MBq/kg	<150 mg/dL	60 minutes	-	3mm/3.27mm		
Vercellino[25]	2012	Gemini Philips	-	5 MBq/kg or 0.135 mCi/kg	-	-	-	-		
