# Supplementary figures and images for: A Systematic Review and Meta-Analysis of the Prognostic Impact of Pretreatment Fluorodeoxyglucose Positron Emission Tomography/Computed Tomography Parameters in Patients with Locally Advanced Cervical Cancer Treated with Concomitant Chemoradiotherapy
Source: Diagnostics (Basel). 2021 Jul 14;11(7):1258. doi: 10.3390/diagnostics11071258 (PMC8304455; doi:10.3390/diagnostics11071258)

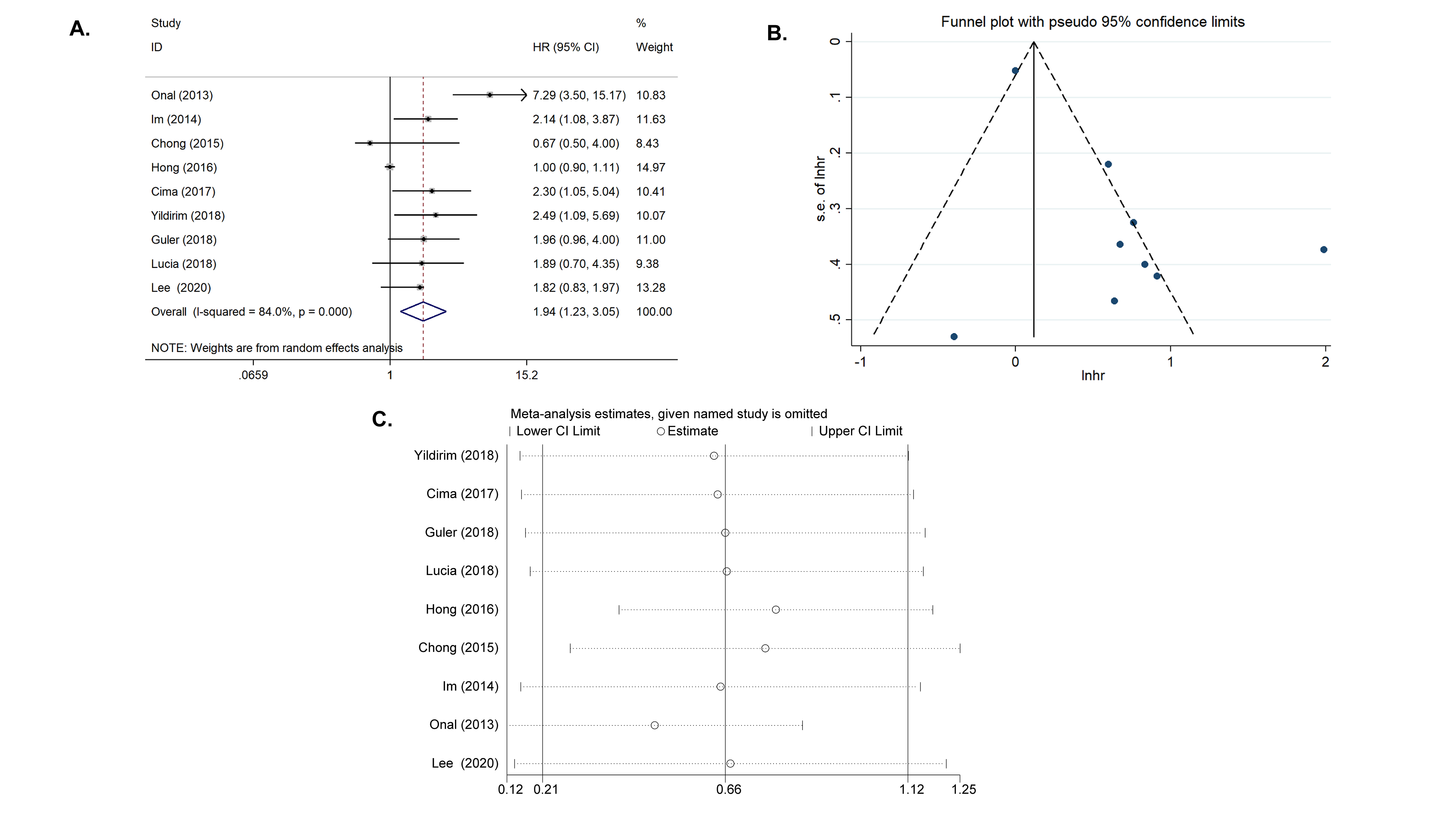

Supplement: Supplementary file 1 [file diagnostics-11-01258-s001.zip › Figure S1.tiff]

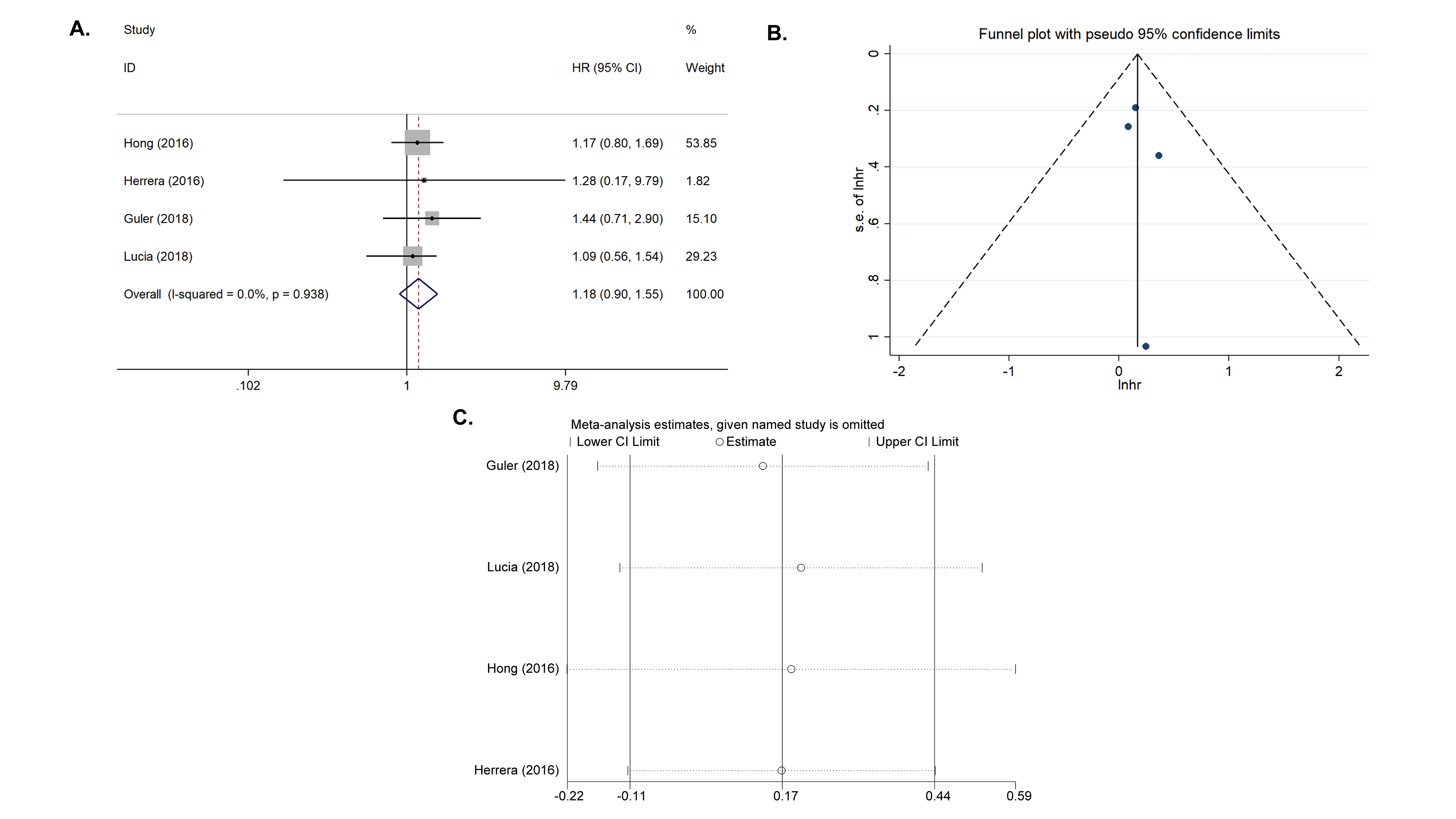

Supplement: Supplementary file 1 [file diagnostics-11-01258-s001.zip › Figure S2.tiff]

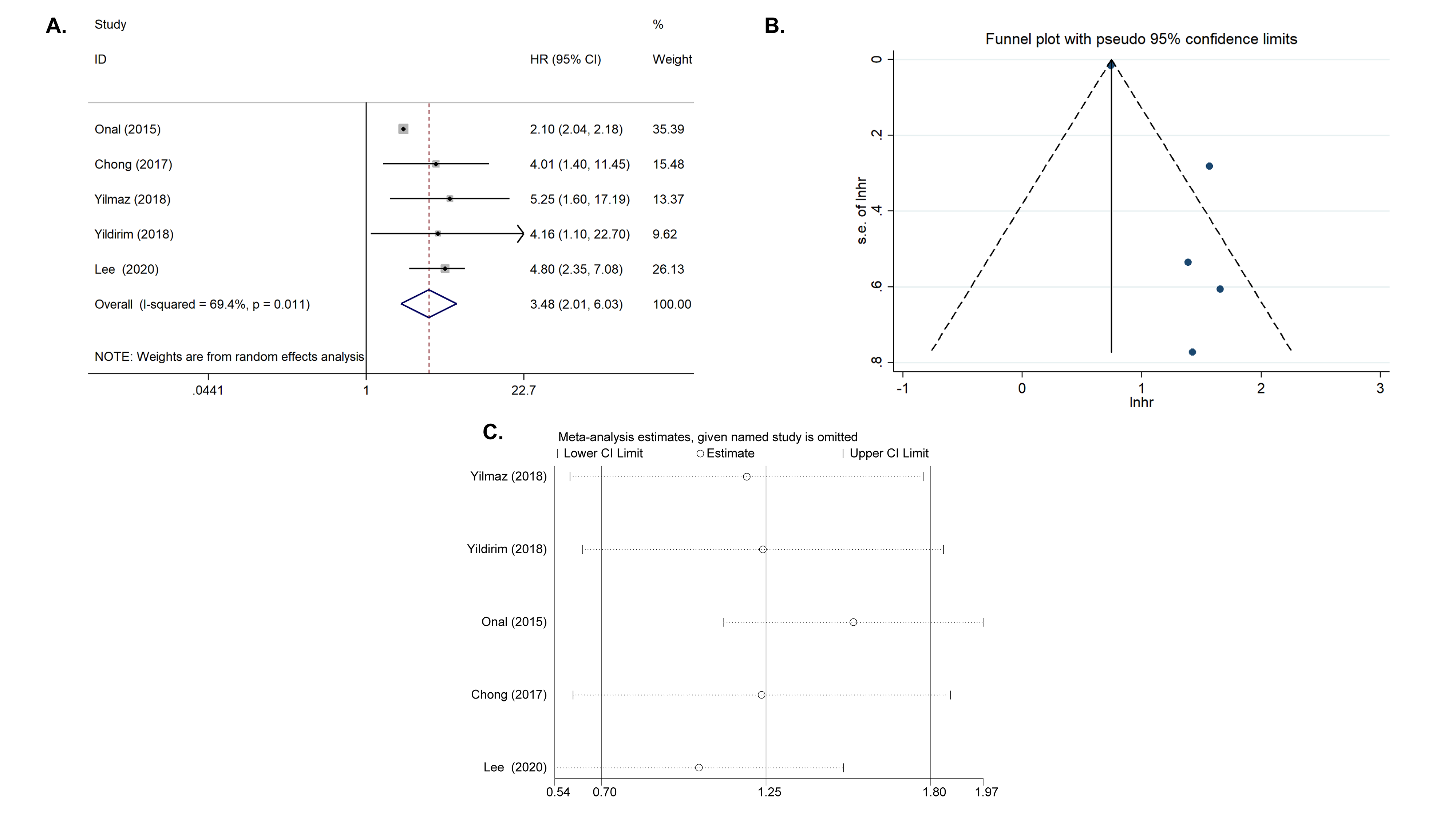

Supplement: Supplementary file 1 [file diagnostics-11-01258-s001.zip › Figure S3.tiff]

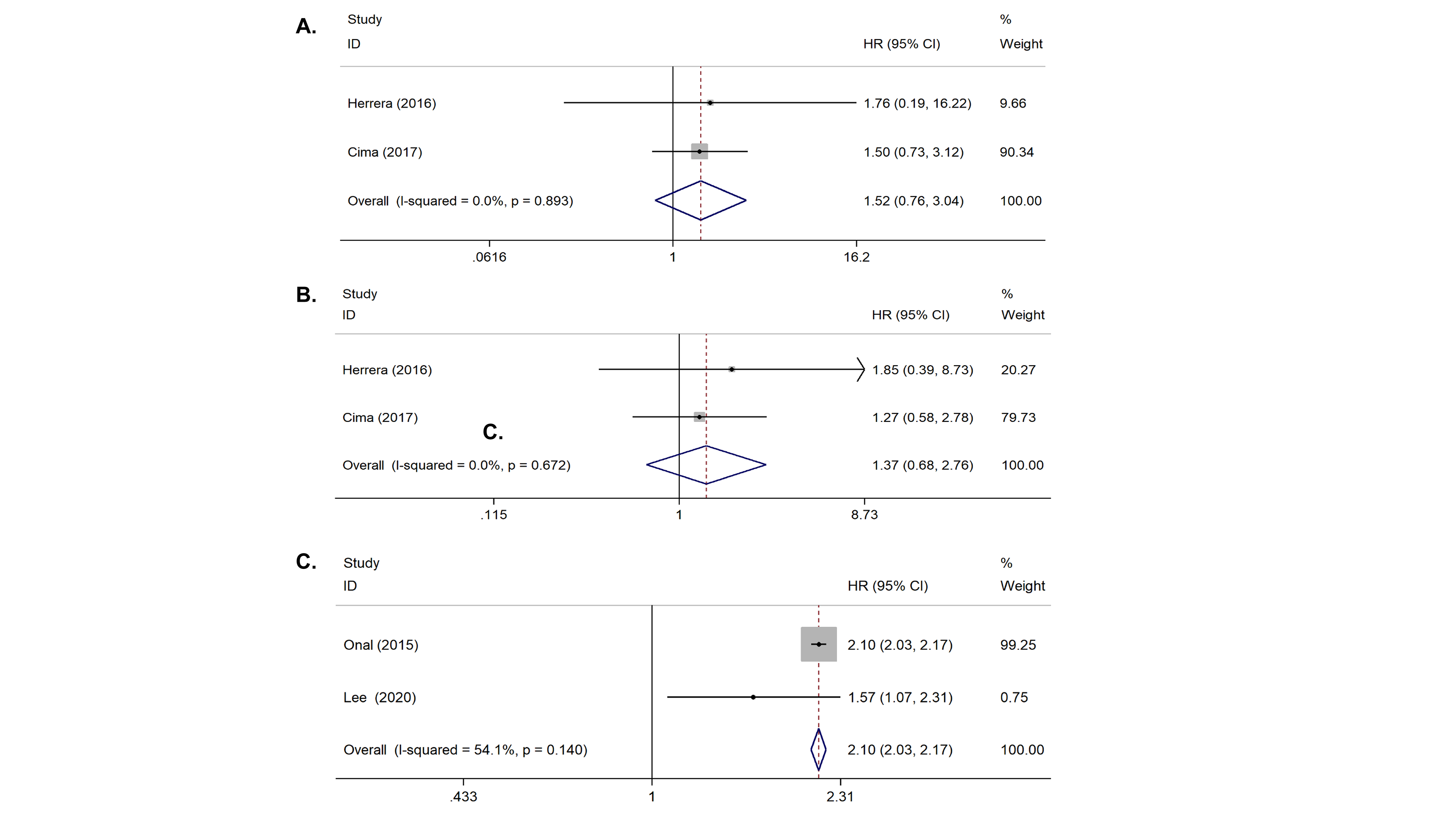

Supplement: Supplementary file 1 [file diagnostics-11-01258-s001.zip › Figure S4.tiff]
